# Development of an automated machine learning-based prediction model and interactive tool for blood transfusion requirements in patients with severe traumatic brain injury

**DOI:** 10.3389/fneur.2026.1757553

**Published:** 2026-04-29

**Authors:** Chunhong Gong, Liangli Chen, Hongxia Chen

**Affiliations:** Department of Blood Transfusion, Xianning Central Hospital, The First Affiliated Hospital of Hubei University of Science and Technology, Xianning, Hubei, China

**Keywords:** automated machine learning, blood transfusion, clinical decision-making, prediction model, severe traumatic brain injury

## Abstract

**Objective:**

To develop an AutoML-based interpretable prediction model for blood transfusion requirements in severe traumatic brain injury (sTBI) patients, optimizing blood resource management through clinical-translational tools.

**Methods:**

In this retrospective cohort study (January 2020–January 2025), 638 sTBI patients were enrolled. Random stratified sampling divided data into training (*n* = 447) and testing (*n* = 191) sets (7:3 ratio). We constructed an Automated Machine Learning (AutoML) framework using the Improved Hannibal Barca Optimizer (IHBO), which synchronously integrated LASSO feature selection verification and Shapley Additive exPlanations (SHAP) interpretability analysis. Model evaluation covered discriminative ability (AUC/PR-AUC), calibration performance (Brier score), and clinical utility (Decision Curve Analysis).

**Results:**

The AutoML model demonstrated exceptional performance in the independent testing set, with ROC-AUC and PR-AUC values reflecting high predictive accuracy. It consistently outperformed comparator models across all metrics, including F1-score (0.8387), while DCA confirmed superior net benefit across clinically relevant thresholds. SHAP analysis identified nine key predictors hierarchically influencing transfusion risk: treatment type, GCS score, INR, K^+^, Ca2^+^, Hct, age, hemorrhagic shock, and skull fracture.

**Conclusion:**

This explainable AutoML framework successfully deciphers multidimensional determinants of sTBI transfusion needs. The clinically deployable interactive system eliminates technical barriers through intuitive nine-feature input, establishing new paradigm for trauma care decision-support and blood resource optimization.

## 1 Introduction

Traumatic Brain Injury (TBI), as one of the primary global causes of disability and mortality, involves a complex chain of clinical decision-making for TBI management (1). Among these, precision in formulating blood transfusion strategies directly correlates with patient survival rates and neurological functional outcomes (2, 3). Within the cohort of severe Traumatic Brain Injury (sTBI) patients, secondary anemia and coagulation disorders are prevalent; however, due to significant heterogeneity in individual injury mechanisms, baseline physiological states, and compensatory capacities, transfusion requirements demonstrate high variability (4). Over-transfusion may induce circulatory overload and immune dysregulation (5), while under-transfusion exacerbates the risk of secondary cerebral hypoxia (6). This clinical dilemma urgently necessitates individualized intervention through objective predictive tools.

Current clinical practices predominantly rely on transfusion decisions based on isolated threshold indicators (e.g., hemoglobin <70 g/L), lacking comprehensive evaluation of patients’ multidimensional pathophysiological status (7, 8). Although artificial intelligence models have made significant strides in medical prediction fields in recent years, existing studies still exhibit three fundamental limitations (9–11): First, model construction often depends on single-center, small-sample cohorts, whose feature weights demonstrate inadequate generalizability across diverse populations, leading to predictive bias; second, most algorithms remain at the “black-box” stage, failing to gain clinical trust due to absence of interpretability verification for prediction logic; third, existing predictive tools frequently require programming environments for operation, imposing excessively high technical barriers for operators. Relevant studies indicate that approximately 76% of clinical prediction models fail clinical translation due to integration challenges with existing diagnostic workflows (12). More notably, current guidelines for blood management in sTBI patients predominantly derive from evidence in non-neurological trauma studies. A customized decision system tailored to the unique pathological mechanisms of craniocerebral trauma—such as lowered thresholds for cerebral oxygen supply–demand imbalance and activation of coagulation-inflammation cascades—remains unestablished (13).

This study, grounded in a translational medicine perspective, aims to bridge the gap between current predictive model research and clinical application. Its core objectives include: constructing a clinically interpretable sTBI transfusion requirement prediction model by integrating adaptive feature engineering and Explainable Artificial Intelligence (XAI) technology to establish prediction logic linking injury mechanisms—physiological reserves—therapeutic interventions; developing a low-threshold interactive clinical decision support tool that transforms the predictive model into a visual system requiring no programming knowledge, enabling clinicians to rapidly assess patient risk and formulate precise transfusion protocols. These innovations not only provide a new paradigm for individualized sTBI treatment but also offer a methodological framework generalizable to other acute critical illness prediction scenarios, demonstrating universal value in advancing smart healthcare implementation in primary hospitals.

## 2 Methods

### 2.1 Study subjects

Our study employed a retrospective cohort design, selecting patients with severe traumatic brain injury admitted to Xianning Central Hospital from January 2020 to January 2025. A total of 638 eligible subjects were enrolled after applying inclusion and exclusion criteria. As a retrospective study, patient informed consent was waived, and the research was approved by the Ethics Committee of Xianning Central Hospital (Ethics Approval No.: K2025-032) in compliance with the Declaration of Helsinki.

*Inclusion criteria*: (1) Diagnosis of traumatic brain injury; (2) Admission Glasgow Coma Scale (GCS) score ≤8; (3) Age ≥18 years.

*Exclusion criteria*: (1) Pregnant women; (2) Transfer to the hospital >24 h post-injury; (3) > 20% missing medical record data.

### 2.2 Data collection

All patient data were extracted from the hospital’s electronic medical record system using structured forms. Two certified researchers independently verified the data quality. Data categories encompassed: (1) Demographic Information and Vital Signs: Age, sex, body temperature, heart rate (HR), systolic pressure (SP), diastolic pressure (DP), shock index (SI = HR/SP), respiratory rate (RR), GCS; (2) Clinical Injury Characteristics: Injury mechanism: Traffic accident, high fall (≥3 m), violent impact, other; Clinical diagnosis: Cerebral contusion, epidural hematoma, subdural hematoma, subarachnoid hemorrhage, brain herniation, axonal injury; Comorbid injuries: Skull fracture (yes/no), other fractures (yes/no), hemorrhagic shock (yes/no); Treatment strategy: Neurosurgery, non-neurosurgical surgery, conservative management; Treatment strategy was prospectively assigned at triage based on clinical indications before transfusion consideration. This captures preoperative planning intent rather than reflecting post-surgical blood loss; (3) Laboratory Indicators: Coagulation profile: Prothrombin time (PT, s), activated partial thromboplastin time (APTT, s), international normalized ratio (INR); Complete blood count: Red blood cell count (RBC, ×10^12^/L), hemoglobin (Hb, g/L), hematocrit (Hct, %), platelet count (PLT, ×10^9^/L); Electrolytes: Na^+^, K^+^, Cl^−^, Ca^2+^ (mmol/L); Blood type: ABO (A/B/O/AB), RhD (positive/negative). All laboratory indicators were strictly obtained from initial venous samples collected during trauma bay admission, prior to any blood product administration or fluid resuscitation exceeding 500 mL crystalloid. This ensured reverse causality from citrate-induced hypocalcemia was avoided; (4) Outcome Indicator: Red blood cell transfusion (yes/no). Where transfusion protocols strictly adhere to national guidelines using hemoglobin thresholds of 70 g/L for non-bleeding patients and 100 g/L for active hemorrhage, with red blood cell units prepared following standardized leukoreduction protocols.

### 2.3 Automated machine learning

First, based on the outcome indicator, the dataset was divided into a training set (*n* = 447) and an independent testing set (*n* = 191) via random stratified sampling at a 7:3 ratio. To prevent data leakage, all subsequent data preprocessing steps were performed exclusively within the training set. For cases ultimately included in the analysis (i.e., those with any variable having a missing rate <20%), we imputed their missing values. Specifically, missing values for continuous variables were imputed using the median of that variable within the training set; missing values for categorical variables were imputed using the mode (most frequent category) within the training set. All imputation parameters (median or mode) were determined on the training set and applied directly to the testing set, strictly simulating the deployment scenario of the prediction model on new data to prevent data leakage. Continuous variables within the training set underwent standardization (*Z*-score normalization), and the fitted scaler was applied to transform the testing set data. We proposed an Automated Machine Learning (AutoML) framework based on the Improved Hannibal Barca Optimizer (IHBO), which simultaneously optimizes key feature selection and hyperparameter tuning for transfusion demand prediction in sTBI patients. The Hannibal Barca Optimizer (HBO) originates from the computational modeling of classical military strategy, employing core tactics including Triple Encirclement Simulation (Cannae Tactic), Perception-based Historical Trend of Frontline, and Parallax Learning to dynamically balance strategic surprise and tactical exploration. These strategies enable it to achieve robust global exploration and local convergence in high-dimensional optimization. IHBO enhanced HBO by improving the initial population distribution through chaotic mapping and adopting a dynamic Lévy flight step strategy to balance exploration and exploitation efficiency. Algorithm performance was validated using the CEC2022 benchmark test function set.

**Figure 1:**
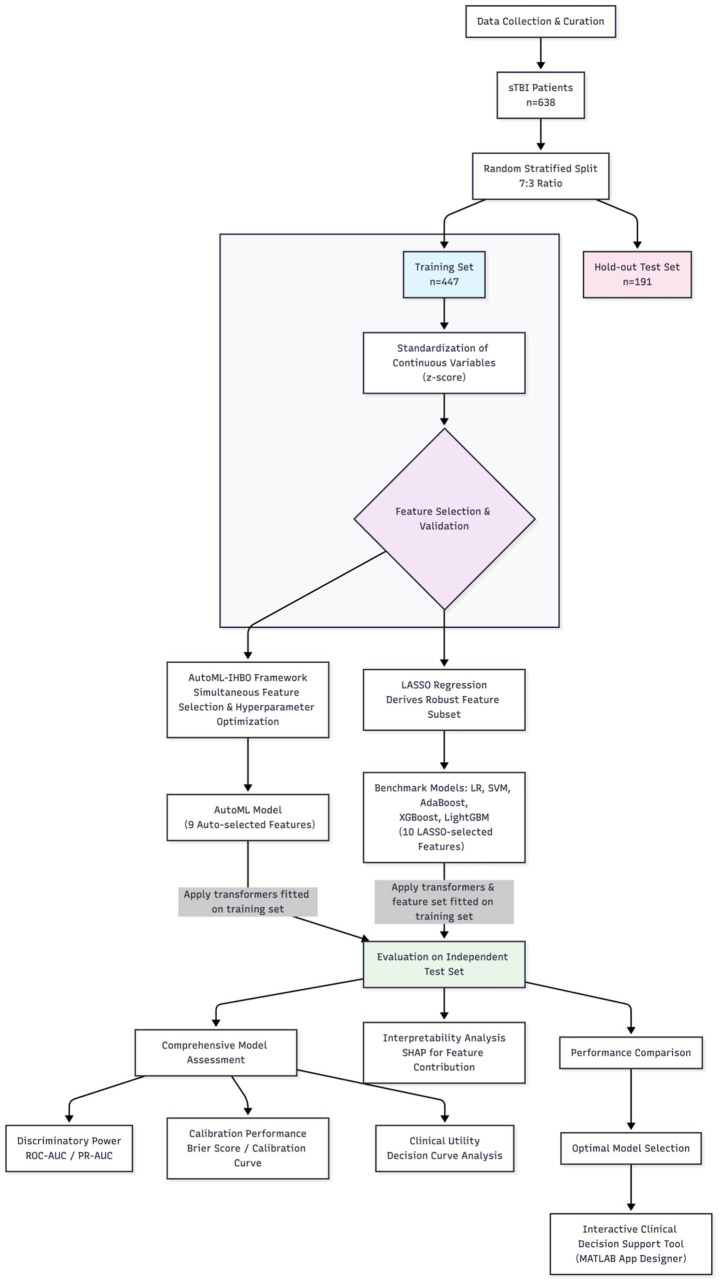
Flow chart of the study.

This framework implements a two-stage optimization: Stage one selects a subset of high-weight features within a discrete space; Stage two fine-tunes hyperparameters within a continuous space (pseudocode see Appendix A). Six comparative models were developed: Logistic Regression (LR), Support Vector Machine (SVM), Adaptive Boosting (AdaBoost), Extreme Gradient Boosting (XGBoost), Light Gradient Boosting Machine (LightGBM), and the proposed AutoML. To ensure fairness and rigor in comparison, all comparative models (LR, SVM, AdaBoost, XGBoost, LightGBM) underwent hyperparameter tuning via Bayesian optimization within predefined search spaces on the training set. Furthermore, to focus the comparison more on the modeling strategies themselves rather than differences in feature sets, these five comparative models were trained and evaluated using a 10-feature subset selected by LASSO regression, which exhibited a 90% overlap rate with the features chosen by AutoML. All models were implemented in MATLAB 2024b. Model training employed 5-fold cross-validation on the training set to mitigate overfitting. The detailed flowchart is shown in Figure 1.

### 2.4 Evaluation metrics

A multidimensional evaluation system was established: (1) Classification Performance: Accuracy (ACC), Sensitivity (SEN), Specificity (SPE), Precision (PRE), F1-score (F1), area under the receiver operating characteristic curve (ROC-AUC), and area under the precision-recall curve (PR-AUC) systematically assessed discriminative ability and stability under class imbalance. (2) Calibration Performance: Calibration curves and Brier score (lower values indicate higher accuracy) evaluated probabilistic prediction calibration. (3) Clinical Application: Decision Curve Analysis (DCA) quantified clinical utility by calculating net benefit (NB) across risk thresholds (pt):


$$\mathrm{NB}=\frac{\mathrm{TP}}{N}-\frac{\mathrm{FP}}{N}\times \frac{p_{t}}{1-p_{t}}$$

(Where *TP*: true positives, *FP*: false positives, *N*: total samples). *NB* was compared against traditional intervention benchmarks to determine effective decision thresholds.

### 2.5 Ablation experiment and comparisons with various AutoML frameworks

To comprehensively evaluate the effectiveness and superiority of the proposed IHBO-AutoML framework, the constructed model’s predictive performance was objectively validated through systematic ablation studies (Ablation Study) and comparative experiments with current mainstream AutoML tools (including TPOT, AutoSklearn, and AutoGluon).

### 2.6 Explainability analysis

After initial feature screening via the AutoML framework, LASSO regression validated feature robustness, and SHapley Additive exPlanations (SHAP) analyzed clinical plausibility. (1) AutoML Feature Screening: Identified prognosis-relevant features within predefined search spaces; (2) LASSO Feature Validation: Applied L1 regularization to the AutoML-selected features to verify sparsity and stability, preventing overfitting; (3) SHAP Analysis: By calculating SHAP values for each feature, quantitative attribution of feature contributions to individual prediction outcomes was performed; comprehensively using summary plots to display global feature importance rankings, with waterfall plots, decision path diagrams, and force plots intuitively presenting the explanatory process of specific prediction cases. This systematically reveals the model’s intrinsic decision-making logic, while SHAP interaction analysis uncovers feature interdependencies.

### 2.7 Interactive tool development

MATLAB App Designer was deployed to build a clinical decision support software. This tool integrates the prediction model, providing clinicians with an intuitive interface for transfusion risk assessment. The system is web-deployable for clinical implementation.

### 2.8 Statistical methods

Data were analyzed in SPSS v26.0. Normality of continuous variables was formally assessed using Shapiro–Wilk tests (*α* = 0.05) supplemented by Q-Q plots prior to selecting appropriate descriptive statistics. Normally distributed variables were expressed as mean ± standard deviation (^−^x ± s), while non-normally distributed variables were summarized as median [interquartile range (IQR)] accordingly. Categorical variables were reported as frequencies and percentages [*n* (%)]. For intergroup comparisons, independent *t*-tests were applied for normal continuous data, Mann–Whitney U tests for non-normal continuous data, and Pearson’s χ^2^ tests for categorical variables. Statistical significance was set at *α* = 0.05 (two-tailed), with all results structured in tables.

## 3 Results

### 3.1 Subject characteristics

The study enrolled 638 patients. The overall sample had a mean age of 55.65 ± 14.87 years, including 442 males (69.28%) and 196 females (30.72%); 296 patients (46.39%) received transfusions. Comparisons of general characteristics between the training and testing sets are presented in Table 1. The results demonstrate balanced distributions across most demographic and clinical baseline characteristics (*p* > 0.05), with statistical differences observed only in isolated variables. These limited differences following random partitioning better reflect the natural variation inherent in real-world data, thereby avoiding potential idealization bias from artificial overmatching. This enhances the generalizability and reliability of our developed model when extrapolated to diverse clinical scenarios.

**Table 1:** Baseline Characteristics of Study Subjects.

Feature	Training set (*n* = 447)	Testing set (*n* = 191)	Statistic	*p*-value
Transfusion received, *n* (%)			χ^2^ = 0.004	0.947
Yes	207 (46.31%)	89 (46.60%)	-	-
No	240 (53.69%)	102 (53.40%)	-	-
Demographics				
Age (years), Median [IQR]	56 [45, 66]	56 [45, 66]	*Z* = −0.017	0.987
Male, *n* (%)			χ^2^ = 0.252	0.616
Yes	307 (68.68%)	135 (70.68%)	-	-
No	140 (31.32%)	56 (29.32%)	-	-
Vital Signs				
Heart rate (beats/min), Mean ± SD	101.07 ± 17.12	101.39 ± 15.50	*t* = −0.236	0.814
Systolic pressure (mmHg), Median [IQR]	130.1 [114.5, 141.5]	130.9 [117.0, 143.6]	*Z* = −0.604	0.546
Diastolic pressure (mmHg), Median [IQR]	77.8 [66.8, 87.0]	77.6 [67.7, 83.7]	*Z* = −0.487	0.626
Shock index, Median [IQR]	0.78 [0.67, 0.92]	0.79 [0.67, 0.91]	*Z* = −0.233	0.816
Respiratory rate (breaths/min), Median [IQR]	21.0 [19.2, 22.6]	20.8 [19.5, 22.3]	*Z* = −0.200	0.841
GCS score, Median [IQR]	7.0 [5.0, 9.0]	7.0 [5.0, 10.0]	*Z* = −2.200	0.028
Clinical diagnosis, *n* (%)			χ^2^ = 10.988	0.052
Cerebral contusion	132 (29.53%)	35 (18.32%)	-	-
Epidural hematoma	54 (12.08%)	25 (13.09%)	-	-
Subdural hematoma	46 (10.29%)	25 (13.09%)	-	-
Subarachnoid hemorrhage	76 (17.00%)	40 (20.94%)	-	-
Brain herniation	79 (17.67%)	44 (23.04%)	-	-
Axonal injury	60 (13.42%)	22 (11.52%)	-	-
Injury mechanism, *n* (%)			χ^2^ = 1.732	0.630
Traffic accident	247 (55.26%)	106 (55.50%)	-	-
High fall (≥3 m)	137 (30.65%)	62 (32.46%)	-	-
Violent impact	55 (12.30%)	22 (11.52%)	-	-
Other	8 (1.79%)	1 (0.52%)	-	-
Treatment strategy, *n* (%)			χ^2^ = 0.059	0.971
Neurosurgery	220 (49.22%)	94 (49.21%)	-	-
Non-neurosurgical surgery	88 (19.69%)	39 (20.42%)	-	-
Conservative management	139 (31.10%)	58 (30.37%)	-	-
Comorbid injuries				
Skull fracture, *n* (%)			χ^2^ = 3.486	0.062
Yes	281 (62.86%)	105 (54.97%)	-	-
No	166 (37.14%)	86 (45.03%)	-	-
Fractures in other locations, *n* (%)			χ^2^ = 6.049	0.014
Yes	211 (47.20%)	70 (36.65%)	-	-
No	236 (52.80%)	121 (63.35%)	-	-
Hemorrhagic shock, *n* (%)			χ^2^ = 0.208	0.649
Yes	45 (10.07%)	17 (8.90%)	-	-
No	402 (89.93%)	174 (91.10%)	-	-
Laboratory indicators				
RBC (×10^12^/L), Median [IQR]	3.74 [3.30, 4.16]	3.71 [3.37, 4.12]	*Z* = −0.340	0.734
Hb (g/L), Median [IQR]	116.5 [99.0, 131.7]	111.6 [97.1, 129.5]	*Z* = −1.388	0.165
Hct (%), Median [IQR]	33.6 [28.8, 38.6]	33.5 [29.1, 37.6]	*Z* = −0.034	0.973
PLT (×10^9^/L), Median [IQR]	140.2 [105.6, 176.2]	141.2 [101.5, 172.1]	*Z* = −0.224	0.823
PT (s), Median [IQR]	13.11 [11.71, 14.46]	13.23 [11.73, 14.60]	*Z* = −0.582	0.561
APTT (s), Median [IQR]	29.13 [24.15, 35.53]	28.81 [24.91, 34.05]	*Z* = −0.467	0.640
INR, Median [IQR]	1.12 [0.99, 1.28]	1.11 [0.95, 1.28]	*Z* = −0.703	0.482
Na + (mmol/L), Median [IQR]	140.2 [137.4, 142.7]	140.0 [137.3, 142.7]	*Z* = −0.229	0.819
K + (mmol/L), Median [IQR]	3.60 [3.26, 3.90]	3.60 [3.32, 3.96]	*Z* = −1.384	0.166
Cl- (mmol/L), Median [IQR]	103.9 [99.2, 108.5]	103.9 [100.1, 108.6]	*Z* = −0.276	0.783
Ca2 + (mmol/L), Mean ± SD	2.07 ± 0.16	2.09 ± 0.16	*t* = −1.489	0.137
ABO, *n* (%)			χ^2^ = 6.082	0.108
ABO-A	121 (27.07%)	66 (34.55%)	-	-
ABO-B	142 (31.77%)	47 (24.61%)	-	-
ABO-O	137 (30.65%)	63 (32.98%)	-	-
ABO-AB	47 (10.51%)	15 (7.85%)	-	-
RhD, *n* (%)			χ^2^ = 0.259	0.611
RhD (+)	440 (98.43%)	189 (98.95%)	-	-
RhD (−)	7 (1.57%)	2 (1.05%)	-	-

### 3.2 Algorithm optimization performance testing

**Figure 2:**
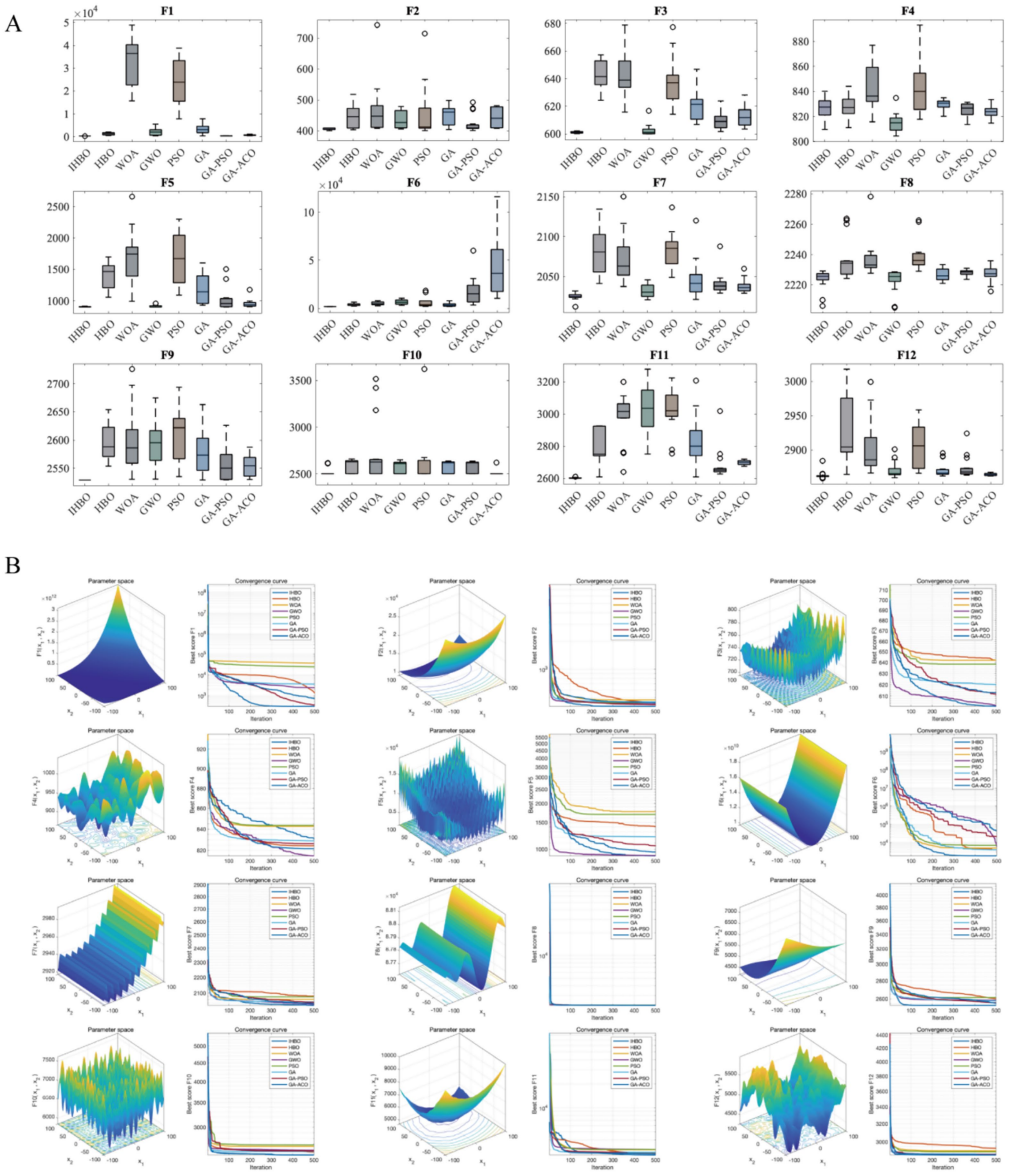
Simulation tests of different algorithms. **(A)** Boxplots of optimization results after 30 independent runs on CEC2022 functions, illustrating algorithm stability and robustness. **(B)** Convergence curves during optimization, reflecting convergence speed and local optima avoidance capability.

To validate the optimization capability of the Improved Hannibal Barca Optimizer (IHBO), comparative tests were performed against HBO, WOA, GWO, PSO, GA, GA-PSO, and GA-ACO algorithms. Experiments utilized all 12 benchmark functions from the CEC2022 test set, with variable dimensions set to 10, population size to 30, and maximum iterations to 500. Each algorithm was independently run 30 times to ensure statistical reliability. Boxplots of the 30 runs demonstrated that IHBO outperformed most algorithms, exhibiting significantly superior stability compared to HBO and others (Figure 2A). Convergence curve analysis further revealed IHBO’s faster convergence speed and lowest risk of local optima (Figure 2B). These results fully confirmed IHBO’s advantages in global optimization and convergence efficiency.

### 3.3 Transfusion demand prediction model construction

**Figure 3:**
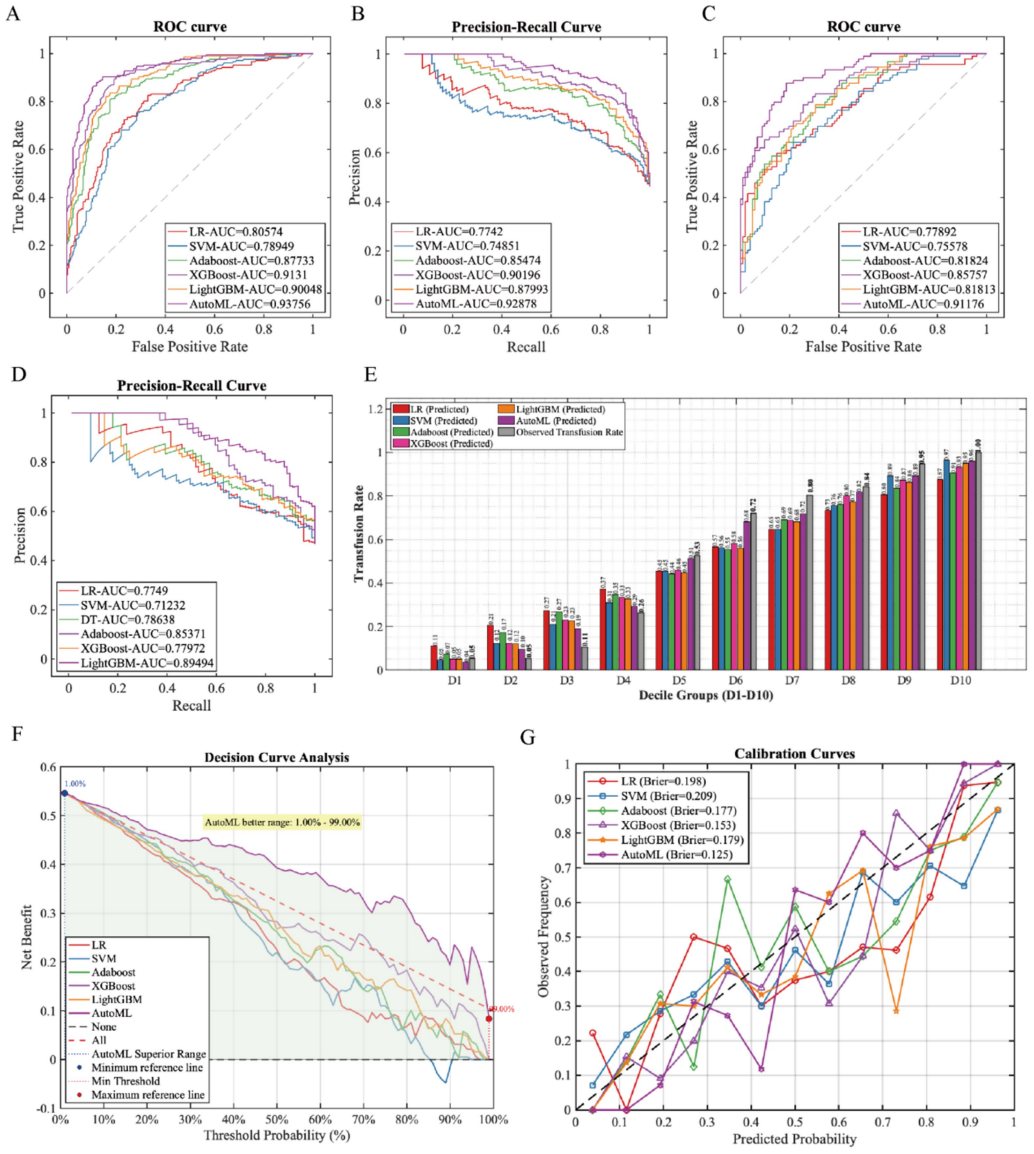
Training and performance validation of prediction models. **(A)** ROC curve (training set); **(B)** PR curve (training set); **(C)** ROC curve (testing set); **(D)** PR curve (testing set); **(E)** comparison of decile-stratified predicted probabilities in the calibration curve; **(F)** DCA curve (testing set); **(G)** calibration curve (testing set).

Six machine learning models were systematically evaluated on the training set using precision, sensitivity, specificity, accuracy, F1-score, and area under the curve metrics. The AutoML model achieved optimal performance across all metrics, with ROC-AUC (0.9376) and PR-AUC (0.9278) (Table 2, Figures 3A,B). Notably, AutoML excelled in F1-score (0.8683), highlighting its clinical utility in balancing precision-recall. The features selected by AutoML were: treatment type, GCS score, INR, Hct, K^+^, Ca^2+^, age, skull fracture, and hemorrhagic shock.

**Table 2:** Performance evaluation metrics of prediction models.

Data set	Models	PRE	SEN	SPE	ACC	F1	ROC-AUC	PR-AUC
Training set	LR	0.6840	0.8261	0.6708	0.7427	0.7484	0.8057	0.7742
	SVM	0.6964	0.7536	0.7167	0.7338	0.7239	0.7895	0.7485
	Adaboost	0.7962	0.8116	0.8208	0.8166	0.8038	0.8773	0.8547
	XGBoost	0.7932	0.9082	0.7958	0.8479	0.8468	0.9131	0.9020
	LightGBM	0.8173	0.8213	0.8417	0.8322	0.8193	0.9005	0.8799
	AutoML	0.8768	0.8599	0.8958	0.8792	0.8683	0.9376	0.9278
Testing set	LR	0.5845	0.9326	0.4216	0.6597	0.7186	0.7789	0.7749
	SVM	0.6048	0.8427	0.5196	0.6702	0.7042	0.7558	0.7123
	Adaboost	0.6452	0.8989	0.5686	0.7225	0.7512	0.8182	0.7864
	XGBoost	0.8507	0.6404	0.9020	0.7801	0.7308	0.8576	0.8537
	LightGBM	0.6931	0.7865	0.6961	0.7382	0.7368	0.8181	0.7797
	AutoML	0.8041	0.8764	0.8137	0.8429	0.8387	0.9118	0.8949

In the testing set, AutoML demonstrated the strongest robustness: ROC-AUC (0.9118) and PR-AUC (0.8949) (Figures 3C,D). Decision Curve Analysis (DCA, Figure 3F) confirmed greater net benefit than traditional methods across 1–99% risk thresholds. The stable net benefit curve indicated excellent generalization. Calibration analysis (Figure 3G) revealed AutoML’s superior calibration performance, with the lowest Brier score (0.112). By comparing decile-stratified predicted probabilities in the calibration curve, AutoML demonstrated a significant advantage (Figure 3E).

### 3.4 Ablation experiment and comparisons with various AutoML frameworks

**Figure 4:**
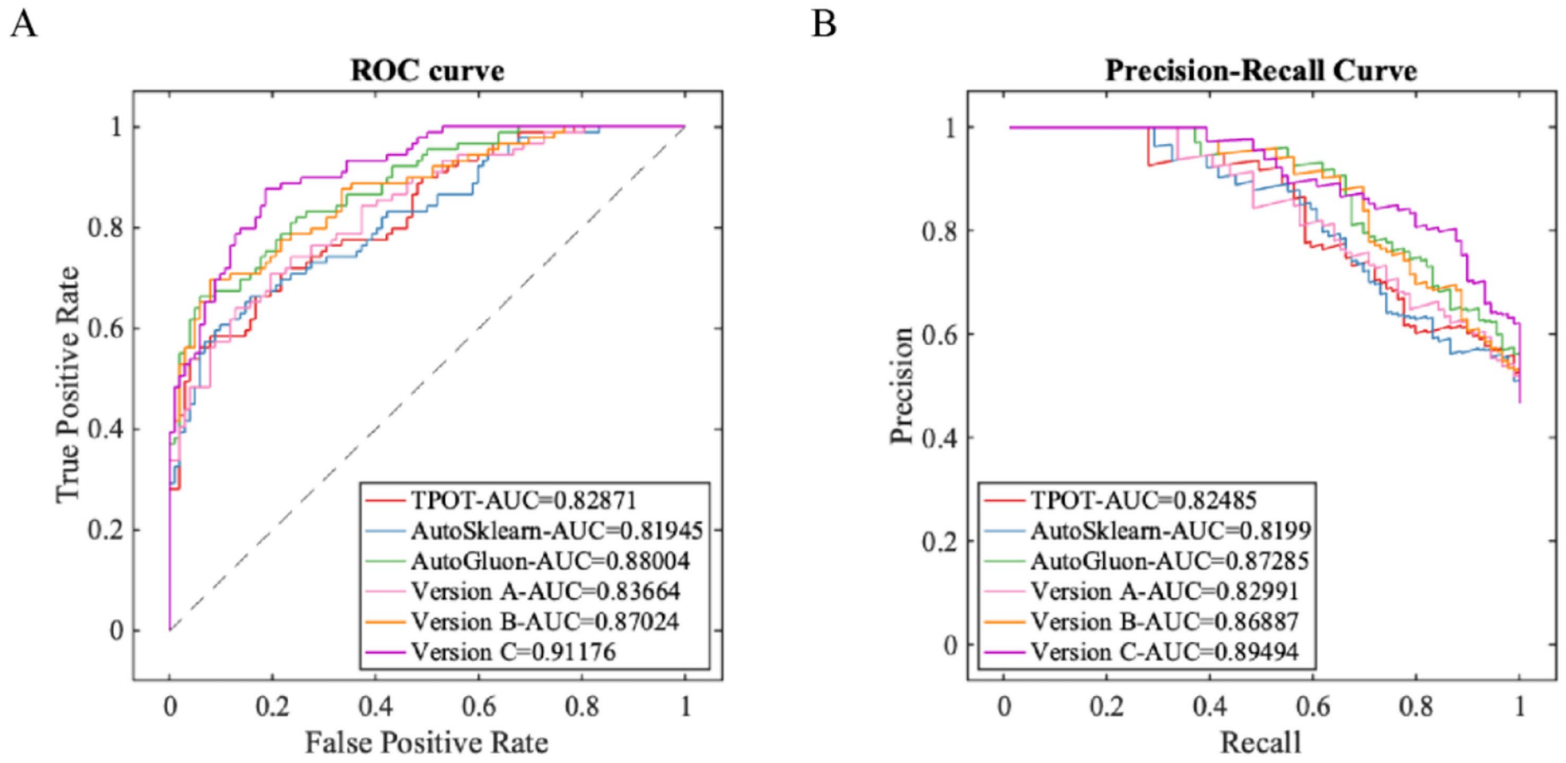
Ablation experiment and AutoML model prediction results. **(A)** ROC curve (testing set); **(B)** PR curve (testing set).

As shown in Table 3 and Figure 4, the proposed full IHBO-AutoML framework (i.e., Version C) achieved optimal or near-optimal performance across most key metrics. It attained an area under the ROC curve (ROC-AUC) of 0.9118, area under the PR curve (PR-AUC) of 0.8949, overall accuracy of 0.8429, and F1-score of 0.8387—all of which significantly outperformed or were comparable to other top competitors. Specifically: The TPOT framework demonstrated superior specificity (0.8824) but lower sensitivity (0.6517), resulting in unremarkable F1-score and AUC metrics. AutoSklearn exhibited extremely high sensitivity (0.9213) but relatively weak precision and specificity, indicating potential over-recall issues. AutoGluon displayed strong overall performance (ROC-AUC = 0.8800), making it the top baseline model; however, it was consistently outperformed by our full model in all metrics.

**Table 3:** Comparisons of ablation experiments and various AutoML results.

Models	PRE	SEN	SPE	ACC	F1	ROC-AUC	PR-AUC
TPOT	0.8286	0.6517	0.8824	0.7749	0.7296	0.8287	0.8249
AutoSklearn	0.7069	0.9213	0.6667	0.7853	0.8000	0.8195	0.8199
AutoGluon	0.8090	0.8090	0.8333	0.8220	0.8090	0.8800	0.8729
Version A	0.7315	0.8876	0.7157	0.7958	0.8020	0.8366	0.8299
Version B	0.9697	0.7191	0.9804	0.8586	0.8258	0.8702	0.8689
Version C	0.8041	0.8764	0.8137	0.8429	0.8387	0.9118	0.8949

The ablation study clearly delineates the individual contributions of two core improvements in the IHBO framework: Version A (using only the standard HBO optimizer for search) achieved mediocre results across multiple metrics (ROC-AUC = 0.8366), indicating limited search efficiency of the baseline optimization strategy. Version B (introducing chaotic mapping-enhanced initial population strategy atop Version A) delivered significant performance gains—precision increased to 0.9697, and specificity reached 0.98046. This validates that enhancing the diversity of optimization starting points effectively guides the search process away from local optima, discovering more discriminative feature subsets and hyperparameter configurations. Version C (the full IHBO integrating chaotic initialization and dynamic Lévy flight search strategy) further balanced exploration and exploitation on the basis of Version B. This markedly improved sensitivity (from 0.7191 to 0.8764) while maintaining high specificity. Consequently, ROC-AUC increased from 0.8702 to 0.9118, and PR-AUC rose from 0.8689 to 0.8949, confirming the dynamic Lévy flight mechanism’s effectiveness in refining local searches during later optimization stages to comprehensively enhance model generalization capability.

### 3.5 Key factor analysis

**Figure 5:**
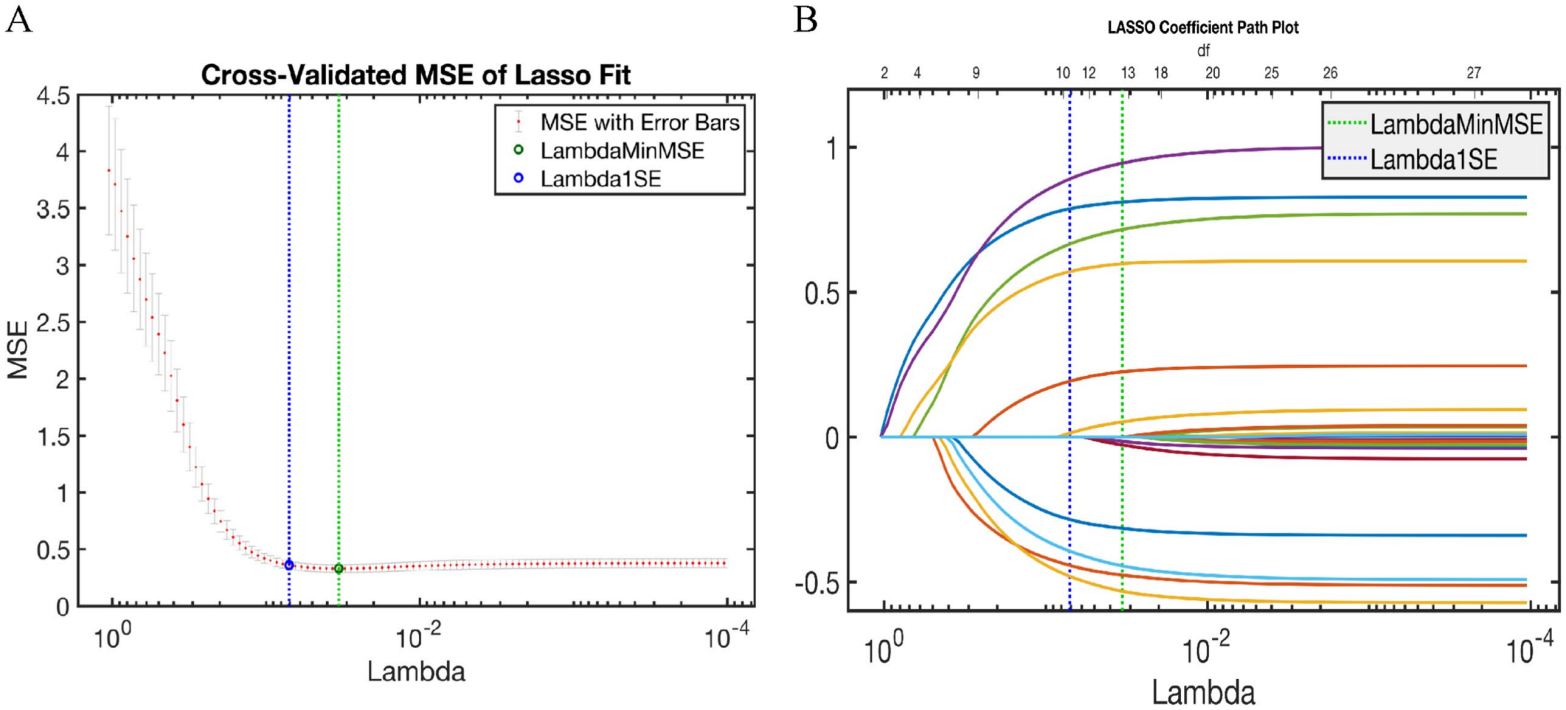
LASSO regression results. **(A)** LASSO trajectory; **(B)** LASSO cross-validation plot.

**Figure 6:**
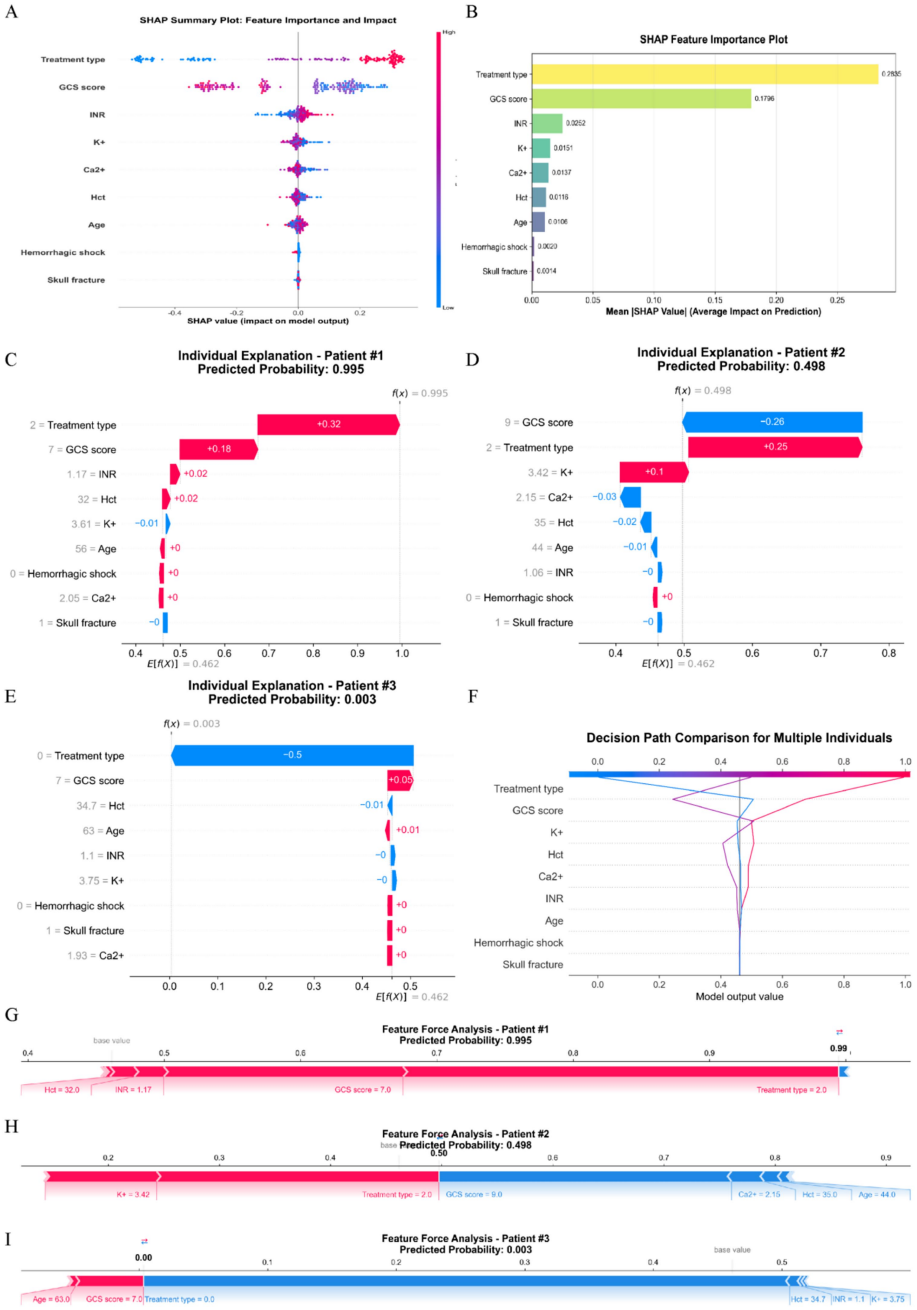
SHAP interpretability analysis. **(A)** The SHAP summary plot; **(B)** the Shapley feature importance plot; **(C–E)** waterfall plots illustrate the cumulative contribution process of each feature to individual patient predictions. The baseline value represents the model’s average prediction for all patients, while feature contributions show how each feature affects the final prediction (red indicating increased risk, blue indicating decreased risk). The sum of all feature contributions yields the final predicted value; **(F)** The decision path plot compares decision pathways across multiple patients, demonstrating how different feature combinations lead to varying prediction outcomes. The horizontal axis shows predicted probabilities, the vertical axis lists features, and the curved pathways trace decision routes from baseline values to final predictions; **(G–I)** Force plots visually demonstrate how each feature “pushes” predictions toward higher or lower risk directions. Red arrows indicate features pushing predictions toward higher risk, blue arrows indicate features pushing toward lower risk, with arrow length representing the magnitude of influence.

(1) *LASSO*: LASSO regression validated AutoML’s feature selection (Figure 5). Variables in the minimal mean squared error (MSE) ± 1 standard error (Lambda1SE) were selected, identifying 10 features: treatment type, GCS score, INR, Hct, K^+^, Ca^2+^, age, skull fracture, hemorrhagic shock, and sex. This included all AutoML-selected features, yielding a 90% overlap (9/10).
(2) *SHAP analysis*: SHAP analysis ranked feature importance as: (1) treatment type, (2) GCS score, (3) INR, (4) K^+^, (5) Ca2^+^, (6) Hct, (7) age, (8) hemorrhagic shock, and (9) skull fracture (Figures 6A,B). By comparing the decision paths of patients at different risk levels (Figure 6F), systematic differences in feature combinations between high-risk and low-risk patients can be observed. The paths of high-risk patients shifted significantly to the right, indicating the synergistic effect of multiple high-risk features.

*Individual interpretations*:

- Case 1 (High-risk, Figures 6C,G): 56 years old, neurosurgery, GCS 7, Hct 32.0%, predicted transfusion probability 99.5%, actual transfusion required. Key risk factors: treatment type (neurosurgery) and GCS score.
- Case 2 (Medium-risk, Figures 6D,H): 44 years old, neurosurgery, GCS 9, all laboratory indices normal, predicted transfusion probability 49.8%, no actual transfusion. The model comprehensively considered treatment type and clinical parameters.
- Case 3 (Low-risk, Figures 6E,I): 63 years old, conservative management, GCS 7, laboratory indices essentially normal, predicted transfusion probability 0.3%, no actual transfusion. The conservative management strategy was the primary low-risk factor.

**Figure 7:**
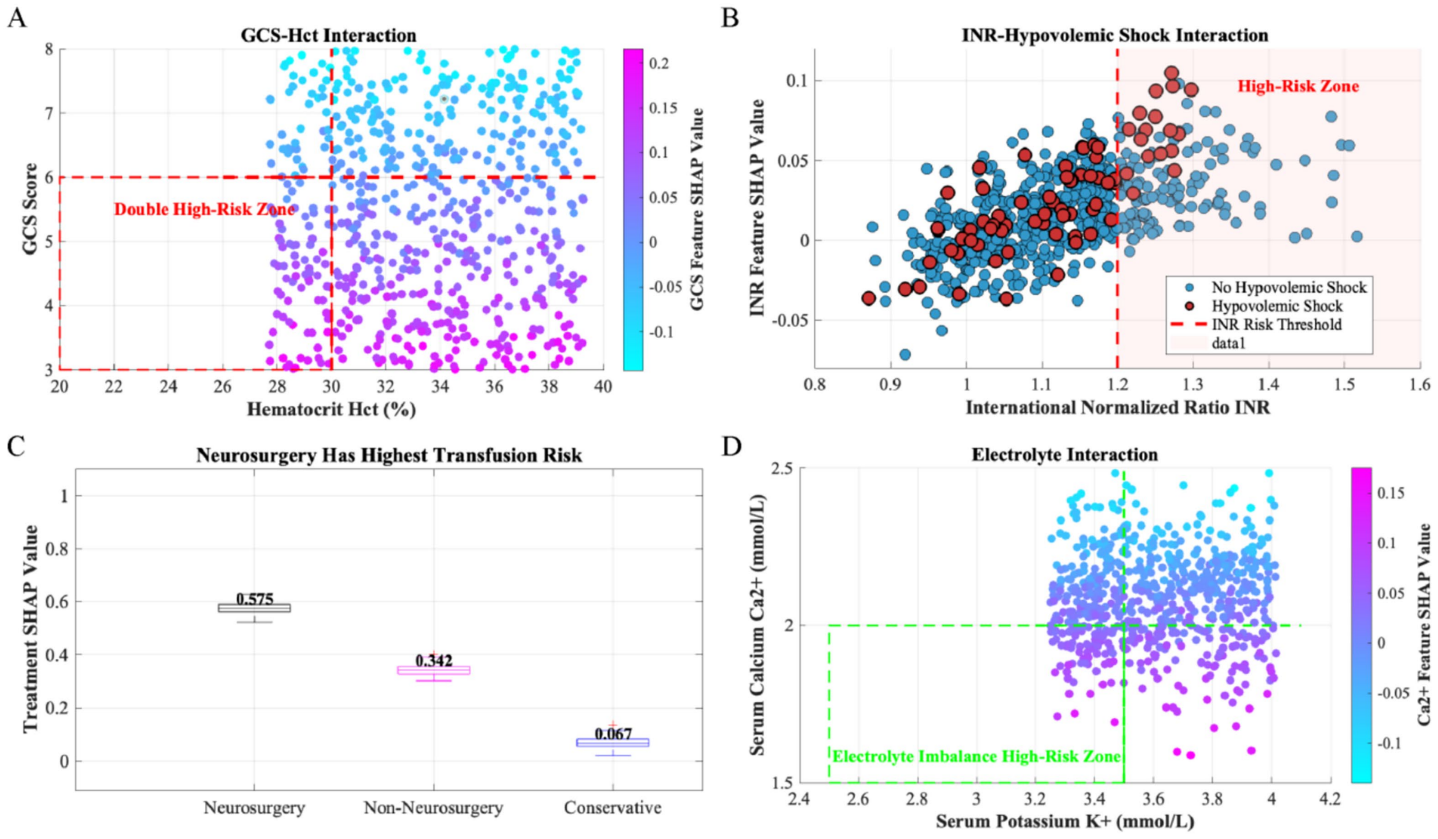
SHAP interaction analyses between key metrics. This study employed SHAP values for feature interaction analysis to identify critical variable interaction patterns in predicting transfusion requirements among patients with severe traumatic brain injury. It must be emphasized that the threshold lines displayed in the figures (e.g., for GCS score, Hct, K^+^, Ca^2+^) serve solely to aid pattern recognition and visualization of interaction effects, and do not represent rigid classification criteria. These thresholds were selected based on widely accepted reference ranges in clinical practice, aiming to facilitate intuitive identification of high-risk zones in scatter plots. However, SHAP analysis fundamentally operates within a continuous multidimensional space—the calculation of interaction effects utilizes the complete continuum of variable information without dependency on any dichotomous thresholds. **(A)** Interaction between GCS score and Hct **(B)** Interaction between INR and Hypovolemic Shock **(C)** SHAP distribution of Treatment Modes **(D)** Interaction between Electrolytes (K^+^ and Ca^2+^).

*Interactions* (Figure 7):

- Interaction between GCS score and Hct: A significant synergistic effect existed between GCS score and Hct. When patients simultaneously exhibited low GCS scores (indicating severe conscious disturbance) and low Hct levels (indicating anemia or blood loss), SHAP values increased markedly, demonstrating a substantial enhancement in predictive contribution for transfusion requirements from this feature combination. The red-labeled area (high-SHAP zone) in the figure concentrated at the junction of low GCS score and low Hct, suggesting significantly increased transfusion risk for comatose patients with anemia.
- Interaction between INR and hypovolemic shock: Hypovolemic shock patients displayed significantly higher INR-feature SHAP values than non-shock patients, indicating that INR plays a more critical predictive role for transfusion requirements when hypovolemic shock is present. This finding suggests that coagulopathy is a key predictor for transfusion needs in hypovolemic shock patients.
- SHAP distribution of treatment modes: Significant differences existed in SHAP distributions across treatment modes (neurosurgery, non-neurosurgical surgery, conservative management). The neurosurgery group exhibited the highest median SHAP value, indicating a stronger association between surgical intervention and transfusion requirements.
- Interaction between electrolytes (K^+^ and Ca^2+^): A synergistic effect existed between serum potassium (K^+^) and serum calcium (Ca^2+^). SHAP values increased substantially when patients simultaneously presented with low K^+^ and low Ca^2+^ levels, suggesting that dual electrolyte abnormalities correlate with increased transfusion risk.

### 3.6 Interactive tool

**Figure 8:**
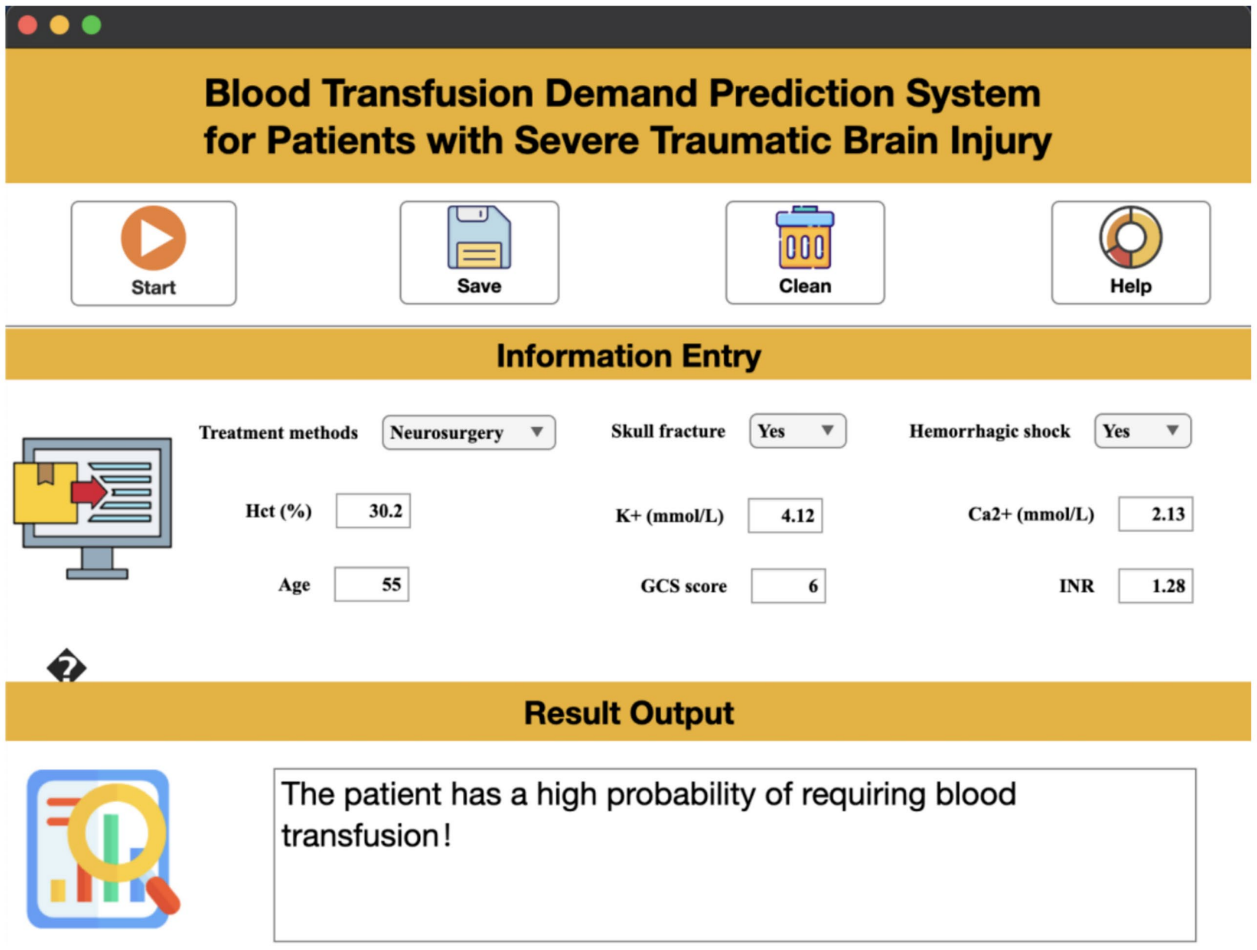
Software system demonstration.

This study identified critical transfusion predictors, but their complex interactions impede intuitive risk assessment in clinical practice. Existing AI methods face high barriers (e.g., programming skills requirement), hindering adoption. We developed an interactive visual system based on the nine key features, offering intuitive operation and clinical utility. Clinicians input only these nine parameters in the ‘Feature Input’ field for instant transfusion demand predictions (Figure 8).

## 4 Discussion

Our study’s AutoML framework and interactive tool represent a significant innovation for predicting blood transfusion needs in severe traumatic brain injury (sTBI) patients. The IHBO-enhanced model demonstrated superior predictive capability by synchronously optimizing feature selection and hyperparameter tuning, addressing algorithmic limitations not fully explored in prior frameworks. For clinical implementation, the MATLAB-based tool eliminated operational barriers, enabling efficient prediction through intuitive input of only nine key features, and validated its net benefit advantage over traditional strategies. Crucially, SHAP explainability analysis revealed an interdependent “core triad” of neurosurgery, coagulopathy (elevated INR), and deep coma (GCS ≤ 6), which clarifies distinctive pathological mechanisms in craniocerebral trauma.

The SHAP-derived feature network highlighted neurosurgery’s primary importance, with significant synergy to deep coma (GCS ≤ 6) and severe anemia (Hct ≤ 30%). Specifically, GCS ≤ 6 indicates brainstem dysfunction, triggering a sympathetic storm that activates coagulation-inflammation cascades: catecholamine release promotes platelet activation and fibrinogen consumption, prolonging INR and inducing trauma-induced coagulopathy (TIC) (14, 15). Neurosurgery amplifies this process—craniotomy disrupts the blood–brain barrier, exposing tissue factor (TF) to activate extrinsic coagulation pathways while surgical blood loss exacerbates anemia, creating a “coagulopathy-anemia” positive feedback loop (14, 16, 17). SHAP interactions underscored this, as synergistic effects intensified transfusion risks; e.g., the risk surge when GCS ≤ 6 coexisted with Hct ≤ 30% reinforces the model’s clinical urgency. Similarly, hypokalemia (K + <3.5 mmol/L) and hypocalcemia (Ca2 + <2.0 mmol/L) constituted high-risk pairs via hypocalcemia suppressing coagulation factor IV-dependent activation and worsening INR derangement, while hypokalemia increased myocardial excitability to destabilize circulation, together reducing vascular tone to induce hypoperfusion. Hemorrhagic shock compounded this, as microcirculatory failure drove tissue hypoxia and acidosis, inhibiting calcium bioavailability (18–20). Skull fractures, reflecting mechanical impact energy, triggered vascular injury through extradural vessel rupture or diploic vein hemorrhage (21). When combined with advanced age, fractures impaired anemia compensation due to diminished hematopoietic reserve and vascular fragility (22, 23), with age-related increases highlighting demographic vulnerability.

While the nine input variables capture critical pathophysiology patterns, we acknowledge practical constraints in resource-limited settings: variables like INR and electrolytes may exhibit delayed availability due to time-intensive laboratory testing (e.g., INR often requires central lab processing with ~30–60-min turnaround). To enhance clinical adoption, future iterations should explore integrating point-of-care testing (POC) for rapid quantification (e.g., handheld INR devices). This tool’s predictive probability threshold (>0.25 for transfusion recommendation) differs from conventional guideline-based approaches (e.g., fixed hematocrit <30% or hemoglobin <7 g/dL) by accounting for multifeature interactions, demonstrating superior net benefit via Decision Curve Analysis and optimized resource allocation. Current guideline thresholds neglect the synergistic impact of neurosurgery, coagulopathy, and deep coma identified in our SHAP analysis.

Despite these advances, three limitations warrant emphasis: First, the single-center retrospective design carries inherent bias. Although random stratified sampling ensured baseline balance between training and testing sets, it cannot account for regional variations in treatment protocols and resources. We acknowledge our data exclusively derives from a tertiary trauma center in central China, where transfusion protocols strictly adhere to national guidelines. While this standardization enhances internal validity, geographic variations in resource allocation (e.g., intraoperative cell salvage availability in 23% of cases at our institution) and injury epidemiology may limit immediate generalizability. We are currently prospectively validating this framework at two additional Level I trauma centers using our institution’s data sharing mechanism. The binary outcome (whether transfusion occurred) defined in this study failed to encompass critical dimensions such as transfusion volume and timing, which are essential for precise blood resource management. Future studies should prioritize collecting prospective, standardized transfusion-related sequential data to develop more advanced models capable of predicting transfusion volume, transfusion frequency, and even optimal transfusion timing points. Second, laboratory data captured only initial admission values, failing to reflect dynamic trajectories of coagulation markers (e.g., INR) or electrolytes (e.g., K^+^, Ca^2+^), thereby limiting time-series predictive modeling. While Treatment strategy’s enhanced preoperative utility, its entanglement with transfusion causality warrants caution. Future studies should stratify transfusion prediction models by treatment modality to isolate intrinsic physiological predictors. Third, technical deployment bottlenecks exist: The MATLAB-based interactive tool presents accessibility limitations in primary hospitals lacking licensed infrastructure, which may exacerbate delays in data acquisition for critical predictors such as INR and electrolytes. Additionally, reliance on these variables creates dependency on laboratory resources that are not universally available in austere environments. Future framework iterations will transition to WebAssembly-based edge computing to reduce operational burdens and support integration with POC devices for low-resource point-of-care use. Future research should focus on: (1) Prospective multicenter cohorts (>2000 cases) with serial measurements of coagulation/electrolyte dynamics to build spatiotemporal predictive models; (2) Lightweight frameworks transitioning to WebAssembly-based edge computing, incorporating radiomics features (e.g., hematoma morphology); (3) Mechanistic exploration via thromboelastography (TEG) to dissect molecular pathways of TIC and investigations into coagulation factor gene polymorphisms (e.g., Factor V Leiden) regulating transfusion needs. These steps will advance the model from a “predictive tool” to a “decision-mechanism dual-driven system.”

## 5 Conclusion

The AutoML framework and interactive tool constructed in this study enable individualized transfusion risk assessment for sTBI patients. By revealing nine key determinants and their interactions via Explainable AI (XAI), this work provides novel insights into the pathophysiological coupling between TIC and secondary brain injury. Clinical deployment of this system promises to transform current extensive transfusion protocols and reduce unnecessary transfusions. More significantly, the methodological framework establishes a new paradigm for intelligent decision-making in critical care, augmenting clinical reasoning with machine intelligence to advance trauma care precision medicine.

## Data Availability

The raw data supporting the conclusions of this article will be made available by the authors, without undue reservation.
