# Supplementary material for: Development of an automated machine learning-based prediction model and interactive tool for blood transfusion requirements in patients with severe traumatic brain injury
Source: Front Neurol. 2026 Apr 29;17:1757553. doi: 10.3389/fneur.2026.1757553 (PMC13167484; doi:10.3389/fneur.2026.1757553)
Supplement: Supplementary file 1 [file Table_1.DOCX]

**Appendix A IBHO AutoML pseudocode**

| PSEUDOCODE 1 |
| --- |
| Algorithm 1: Workflow of the IHBO-AutoML Collaborative Optimization Framework  Input: Training dataset D_train, Base learner set L = {LR, SVM, RF, XGBoost, LightGBM},  Complete feature set F, Maximum iterations T, Population size N  Output: Optimal model M*, Optimal feature subset F*, Optimal hyperparameter configuration θ*  // === Phase 1: Enhanced Initialization via Chaotic Mapping ===  1: Initialize the Improved Hannibal Barca Optimizer (IHBO)  2: P ← ∅ // Initialize an empty population  3: for i = 1 to N do  4: // **Improvement 1: Chaotic Mapping for Initial Population Distribution**  5: // Generate a chaotic sequence value ‘c’ using Logistic/Tent map for better uniformity and diversity  6: c ← ChaoticMap(i) // e.g., Logistic Map: x_{n+1} = μ * x_n * (1 - x_n)  7:  8: // Encode an individual ‘p’ using chaotic-driven randomization  9: p.feature_mask ← GenerateBinaryMask(F, chaos_seed=c) // \|F\|-dim 0-1 vector for feature selection  10: p.learner_id ← SelectLearnerIndex(L, chaos_seed=c) // Integer index selecting from L  11: p.hyperparams ← InitializeHyperparams(L[p.learner_id], chaos_seed=c) // Real-valued vector  12: p.fitness ← -∞ // Initialize fitness  13: P ← P ∪ {p}  14: end for  // === Phase 2: Collaborative Optimization Loop with Dynamic Lévy Flight ===  15: for t = 1 to T do  16: for each individual p in population P do  17:  18: // === Step A: AutoML Pipeline - Model Construction & Evaluation ===  19: F_subset ← ApplyMask(F, p.feature_mask) // Decode feature subset  20: L_selected ← L[p.learner_id] // Decode selected learner  21: θ ← DecodeHyperparams(p.hyperparams, L_selected) // Decode hyperparameters  22:  23: // Core AutoML Process: Instantiate, train, and evaluate the model  24: M_candidate ← AutoML.Pipeline(  25: learner = L_selected,  26: features = F_subset,  27: hyperparameters = θ,  28: data = D_train,  29: validation = "5-fold CV" // Internal validation for fitness evaluation  30: )  31:  32: // Performance as Fitness (Primary: AUC)  33: p.fitness ← M_candidate.evaluate_cv_auc()  34:  35: // Update global best solution  36: if p.fitness > best_fitness then  37: best_fitness ← p.fitness  38: M*_temp ← M_candidate  39: F*_temp ← F_subset  40: θ*_temp ← θ  41: end if  42:  43: end for  44:  45: // === Step B: IHBO Population Evolution with Dynamic Lévy Flight ===  46: // **Improvement 2: Dynamic Lévy Flight for Balancing Exploration and Exploitation**  47: Calculate current iteration ratio: α = t / T  48: for each individual p in P do  49: // Select a guiding individual (e.g., global best or a random elite)  50: p_guide ← SelectGuideIndividual(P, strategy="global_best")  51:  52: // **Core Update Formula with Dynamic Lévy Step**  53: // 1. Compute base displacement influenced by the guide  54: base_step ← β ⊗ (p_guide.position - p.position) // β is a control parameter  55:  56: // 2. Generate dynamic Lévy flight step length, weighted by exploration factor (1-α)  57: levy_step_length ← LevyFlight(β=1.5) * (1 - α + ε) // ε is a small constant  58: // As ‘α’ increases from 0 to 1, the step length decays, shifting focus from exploration to exploitation  59:  60: // 3. Update the encoded continuous hyperparameter vector  61: p.hyperparams ← p.hyperparams + base_step + levy_step_length ⊙ randn(dim)  62:  63: // 4. Update binary feature mask via a sigmoid transfer function  64: for each gene in p.feature_mask do  65: probability ← Sigmoid(p.hyperparams_corresponding[gene])  66: p.feature_mask[gene] ← (rand() < probability) ? 1 : 0  67: end for  68:  69: // Optional: Apply boundary checks and repair mechanisms  70: p.hyperparams ← ClipToBounds(p.hyperparams, L_selected)  71: end for  72:  73: // (Optional) Apply a local refinement (e.g., simplex search) to the global best individual every K iterations  74: if t % K == 0 then  75: P[best_index].hyperparams ← LocalRefinement(P[best_index].hyperparams, D_train)  76: end if  77:  78: end for  // === Phase 3: Final Model Refitting & Output ===  79: // Re-train the optimal configuration on the entire training set  80: M* ← AutoML.Pipeline(learner=L_selected*, features=F*_temp, hyperparameters=θ*_temp, data=D_train, validation="none")  81: F* ← F*_temp  82: θ* ← θ*_temp  83:  84: // The final model M* is then evaluated on a strictly held-out independent test set  85: return M*, F*, θ* |
